# Structural, Morphological, Electrical and Electrochemical Properties of PVA: CS-Based Proton-Conducting Polymer Blend Electrolytes

**DOI:** 10.3390/membranes10040071

**Published:** 2020-04-15

**Authors:** Ayub Shahab Marf, Ranjdar M. Abdullah, Shujahadeen B. Aziz

**Affiliations:** 1 Advanced Polymeric Materials Research Lab., Department of Physics, College of Science, University of Sulaimani, Qlyasan Street, Sulaimani 46001, Iraq; ayub.shahab@gmail.com (A.S.M.); ranjdar.abdullah@univsul.edu.iq (R.M.A.); 2 Department of Civil Engineering, College of Engineering, Komar University of Science and Technology, Sulaimani 46001, Iraq

**Keywords:** PVA:CS polymer blend, NH_4_I salt, XRD and FESEM, impedance, dielectric properties, TNM and LSV study

## Abstract

Polymer blend electrolytes based on poly(vinyl alcohol):chitosan (PVA:CS) incorporated with various quantities of ammonium iodide were prepared and characterized using a range of electrochemical, structural and microscopic techniques. In the structural analysis, X-ray diffraction (XRD) was used to confirm the buildup of the amorphous phase. To reveal the effect of dopant addition on structural changes, field-emission scanning electron microscope (FESEM) was used. The protrusions of salt aggregates with large quantity were seen at the surface of the formed films at 50 wt.% of the added salt. The nature of the relationship between conductivity and dielectric properties was shown using electrochemical impedance spectroscopy (EIS). The EIS spectra were fitted with electrical equivalent circuits (EECs). It was observed that both dielectric constant and dielectric loss were high in the low-frequency region. For all samples, loss tangent and electric modulus plots were analyzed to become familiar with the relaxation behavior. Linear sweep voltammetry (LSV) and transference number measurement (TNM) were recorded. A relatively high cut-off potential for the polymer electrolyte was obtained at 1.33 V and both values of the transference number for ion (t_ion_) and electronic (t_elec_) showed the ion dominant as charge carrier species. The TNM and LSV measurements indicate the suitability of the samples for energy storage application if their conductivity can be more enhanced.

## 1. Introduction

New materials that follow green chemistry principles have been focused upon extensively due to the lower release of pollutants into the environment [1]. Two strong alternatives, namely batteries and supercapacitors as energy storage devices, appear to replace other non-sustainable energy sources, such as oil, nuclear fuel and other fossil fuels [2,3,4]. Solid polymer electrolytes (SPEs) have been extensively studied to examine their application to large-scale production, within capacitor systems [5]. Polymer electrolytes are membranes for the ion transport mechanism. For example, a poly(vinyl alcohol) (PVA) polymer host is as assumed to be one of the cornerstones for the preparation of various types of polymer electrolytes, because of its capability of dissolving a number of inorganic salts and also its biodegradability [6,7]. On the other hand, composites and natural polyphenols are crucial for membrane separation technology and the treatment of wastewater dyes [8,9]. PVA is mainly composed of vinyl alcohol groups (i.e., enriched with polar oxygen atoms) that can make complexes with the salt cations, thus forming polymer electrolyte complexes. Furthermore, PVA is characterized by several inherent properties, for instance, non-toxicity, affordability, a relatively high capacity of charge storage and exceptional mechanical properties [10,11]. The ability of PVA to form electrolytes is associated with its hydrophilic properties [12] and several hydroxyl groups attached to methane carbons (CH_2_-CH) on the PVA backbone [13]. However, natural biopolymers have been considered as strong candidates in the preparation of polymer electrolytes for long-term usage, due to their biodegradability. In addition, natural polymers are relatively cost-effective and environmentally friendly, exhibit high solubility and are able to form films with desired shapes [1]. The most popular natural polymers are chitosan, starch and cellulose that have drawn the attention of many research groups [5]. In terms of structure, chitosan (CS) is a cationic polysaccharide within which β-1–4-linked 2-amino-2 deoxy-d-glucopyranose repeats in a sequence of a billion units. It is easily obtained from the alkaline N-acetylation reaction of chitin. This natural polymer is superior over polymers in terms of biodegradability, biocompatibility, low toxicity and affordability [14]. The presence of amino groups on the CS backbone makes this natural polymer into an exceptional biopolymer. This enables CS to create ion-conducting polymer electrolytes [15]. The amine groups within the CS structure act as electron-rich donors that interact with alkaline metal salts. Based on these facts, CS meets the requirements as a host polymer that encompasses ions using the salt solvation process [15,16]. Moreover, CS has shown membrane properties, such as low methanol permeability, the existence of functional groups within the backbone and hydrophilicity that makes CS practical in a relatively low humidity and high temperature environment [17].

The problem of PVA’s low conductivity has motivated researchers to carry out studies to solve this issue. Kim et al. investigated PVA and lithium trifluoromethane sulfonate (LiCF_3_SO_3_, LiTf) salt in an attempt to improve the low ionic conductivity. It was found that the ionic conductivity of the PVA-based SPE could be increased with increasing salt concentration [18]. It has also been emphasized that the polymer blending approach is an appropriate way to increase ionic conductivity as a result of lowering the degree of crystallinity [19]. In this methodology, two or more polymers can be combined with or without forming primary chemical bonds, such as ionic and covalent bonds [17]. A modification of PVA’s crystalline phase was achieved through chitosan polymer blending. The most common additives used in the preparation of proton (H^+^)-conducting SPEs are strong inorganic acids (H_3_PO_4_ and H_2_SO_4_) and ammonium salts. It is important to mention that SPEs containing inorganic acids usually suffer from chemical degradation that makes them practically unusable [20,21]. Alternatively, proton-conducting SPEs having relatively high thermal stability and ionic conductivity can be achieved using ammonium salts. To know the degree of dissociation of ammonium salts, it is important to take into consideration the lattice energy. The lattice energy of a number of ammonium salts is given as follows: ammonium chloride (NH_4_Cl), ammonium bromide (NH_4_Br) and ammonium iodide (NH_4_I) possess a lattice energy of 698 kJ/mol, 665.3 kJ/mol, and 634 kJ/mol, respectively [22,23]. Based on lattice energy, a biopolymer electrolyte can be enriched with ions using ammonium iodide (NH_4_I) [24].

In condensed matter physics, ion conduction and dielectric relaxation in solid materials are two hot topics that are under intensive study. In particular, dealing with dielectric relaxation in SPEs has been found to be an appropriate way to obtain insight into the characteristics of cation–polymer interactions. This is because the dielectric constant is a measure of a polymer material’s ability to dissolve inorganic salts [25,26].

The present work is aimed at studying PVA:CS systems incorporated with various quantities of NH_4_I, examined through electrochemical impedance spectroscopy (EIS), X-ray diffraction (XRD) and scanning electron microscopy (SEM). The results of the present work are crucial to understanding the basic relationships between DC conductivity and dielectric properties. The study of tanδ and electric modulus is informative about the ion conduction mechanism in polymer electrolytes. On the other hand, the electrochemical investigations (TNM and LSV) indicate the suitability of the samples for electrochemical device application if their conductivity is enhanced using various approaches such as the addition of fillers or plasticizers. In future works, we will consider these approaches to improve the conductivity. From linear sweep voltammetry (LSV), the PVA:CS biopolymer electrolyte in this work was found to be electrochemically stable up to 1.33 V, which is important for use in electrical double-layer capacitor (EDLC) applications. 

## 2. Experimental Details

### 2.1. Materials and Sample Preparation

All the chemicals were purchased from commercial suppliers (Sigma-Aldrich and Merck) and used as received. The chitosan (CS) with medium molecular mass (CAS 9012-76-4) was obtained from Sigma-Aldrich (Sigma-Aldrich, Warrington, PA, USA), and poly(vinyl alcohol) (PVA) and ammonium iodide (NH_4_I) were purchased from Merck. All these materials were used as raw materials in the synthesis of the PVA:CS electrolyte systems. The blend polymer electrolyte synthesis procedure involves dissolving 0.5 g of CS in 30 mLof a 1 wt.% acetic acid solution under magnetic stirring continuously for several hours. Meanwhile, 0.5 g of PVA was dissolved in 20 mLof distilled water at 90 °C. This PVA solution was left to cool down to ambient temperature. Afterwards, the PVA solution and CS were mixed with a magnetic stirrer under continuous stirring until a homogenous solution was obtained. Consequently, different wt.% amounts of NH_4_I were added to the final solution. The salt addition was varied from 10 to 50 wt.%, and this series of sample solutions was coded as PVCS1, PVCS2, PVCS3, PVCS4 and PVCS5. The coded solutions were incorporated with 50% CS and 50% PVA integrated with 10, 20, 30, 40 and 50 wt.% amounts of ammonium iodide (NH_4_I), respectively. The solution mixtures were then poured into differently labeled Petri dishes and left to evaporate slowly at room temperature for a couple of weeks to form solvent-free films. Finally, for further drying, the films were put into desiccators that were filled with blue silica gel desiccant to ensure the removal of any trace amount of solvent or moisture. Table 1 summarizes the designation and composition of the various PVA:CS:NH_4_I polymer blend electrolytes.

### 2.2. SEM and XRD Study

The surface morphology images of the prepared blend electrolyte films were acquired using SEM (FEI Quanta 200 FESEM). The structural texture study involved recording X-ray diffraction (XRD) patterns at room temperature using an X-ray diffractometer (Bruker AXS) with operating current and voltage of 40 mA and 40 kV, respectively.

### 2.3. Electrical Impedance Spectroscopy (EIS)

Impedance analyses of the prepared samples were performed using an LCR meter (HIOKI 3531 Z HiTester, Nagano, Japan), connected to a computer. Prior to taking measurements, the prepared films were cut into small discs of 2 cm diameter and sandwiched between two stainless steel electrodes with the aid of spring clips to ensure a good contact. The measurements were conducted within the frequency range of 50 Hz to 1 MHz at ambient temperature. The bulk resistance (*R_b_*) was obtained from the intercept of the plot with the *Z′* axis. Both real and imaginary parts of the complex permittivity (ε*) and the complex modulus (M*) as well as tanδwere extracted from the impedance data (i.e., *Z′* and *Z″*) using the following equations [27,28]:

(1) $$\varepsilon {}^{\prime }=\frac{Z{}^{\prime \prime }}{\omega C_{0}\left({Z^{\prime }}^{2}+Z^{\prime \prime }{}^{2}\right)}$$

(2) $$\varepsilon {}^{\prime \prime }=\frac{Z{}^{\prime }}{\omega C_{0}\left({Z^{\prime }}^{2}+{Z^{\prime \prime }}^{2}\right)}$$

(3) $$\tan\delta =\frac{Z^{\prime }}{Z^{\prime \prime }}$$

(4) $$M{}^{\prime }=\frac{\varepsilon {}^{\prime }}{\left({\varepsilon^{\prime }}^{2}+{\varepsilon^{\prime \prime }}^{2}\right)}=\omega C_{0}Z{}^{\prime \prime }$$

(5) $$M{}^{\prime \prime }=\frac{\varepsilon \prime \prime }{\left({\varepsilon^{\prime }}^{2}+{\varepsilon^{\prime \prime }}^{2}\right)}=\omega C_{0}Z{}^{\prime }$$

where *ω* is the angular frequency of the applied field (*ω* = *2πf*) and *ε*′ and *ε*″ are the dielectric constant and dielectric loss, respectively. The real and imaginary parts of the complex modulus M* are represented by *M*′ and *M*″, respectively. The *C_o_* is the vacuum capacitance given by *ε_0_A/t,* where *ε_o_* is a permittivity of free space, *A* is the electrode cross sectional area and *t* is the film thickness.

### 2.4. Transference Number Measurement (TNM)

In this work, both ionic (*t_ion_*) and electronic (*t_el_*) transference numbers were obtained from the cell polarization versus time at room temperature. Two stainless steel (SS) electrodes were used for the conducting sample of the SPE. The cell was polarized at a working voltage of 0.20 V. For this purpose, a V&A Instrument DP3003 digital DC power supply was used at room temperature.

### 2.5. Linear Sweep Voltammetry (LSV) Study

Linear sweep voltammetry (LSV) was recorded for SPEs to determine the electrochemical stability by measuring the oxidation decomposition voltage at room temperature. The LSV recording was carried out in the cell of two electrodes of stainless steel electrodes where the film sample was kept using a Digi-IVY DY2300 potentiostat (10 mV/s scan rate).

## 3. Results and Discussion

### 3.1. Structural Study

**Figure 1:**
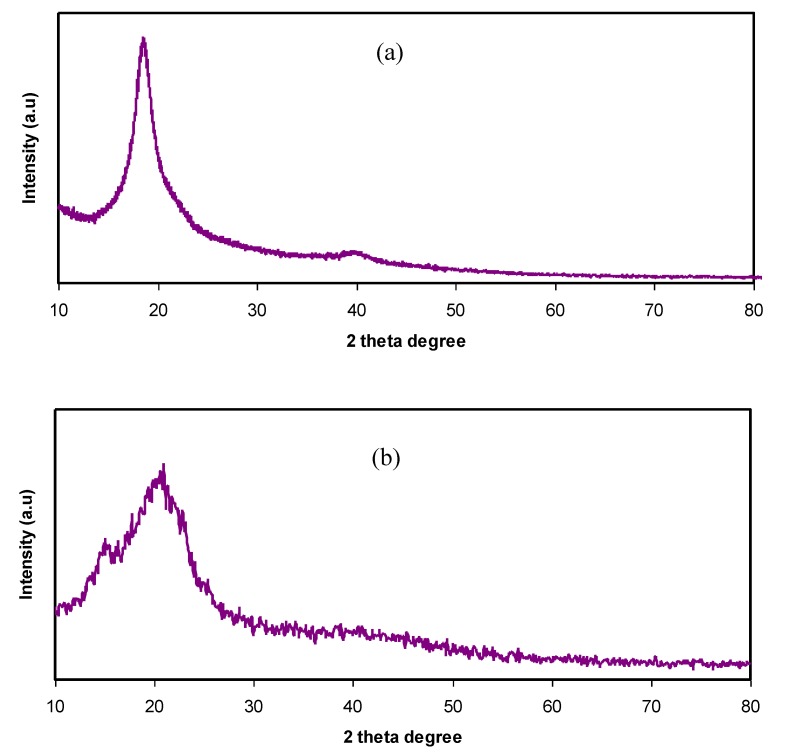
XRD pattern for (**a**) pure poly(vinyl alcohol) (PVA) and (**b**) pure chitosan (CS).

The XRD patterns of pure PVA and pure CS are presented in Figure 1a,b, respectively. In Figure 1a, a peak appears around 2θ = 18°, indicating the semi-crystalline behavior of pure PVA [11]. This is due to the existence of OH groups along the main chain of PVA, providing a relatively strong intermolecular and intramolecular hydrogen bonding. At the same time, a broad peak centered at 2θ = 40.7° refers to amorphous phases in the PVA structure.

**Figure 2:**
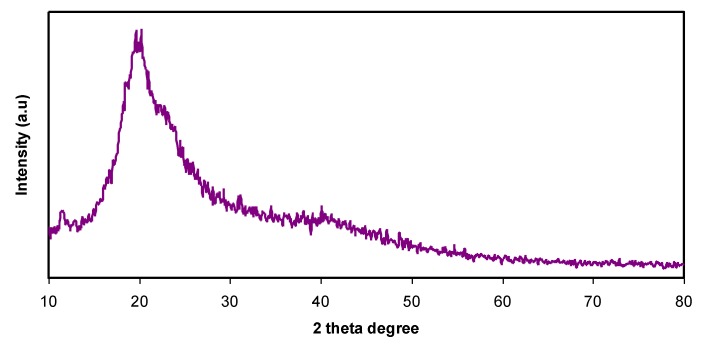
XRD pattern for PVA:CS [50:50] polymer blend films.

**Figure 3:**
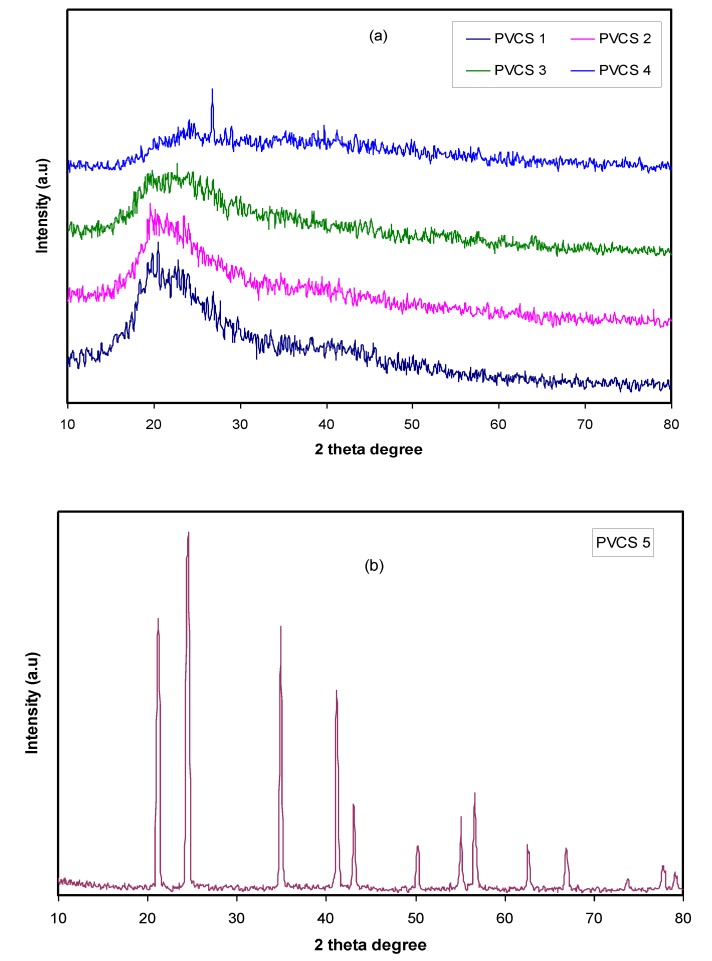
XRD pattern for PVA:CS [50:50] polymer blend electrolytes (**a**) from 10 to 40 wt.% of NH_4_I and (**b**) for 50 wt.% of NH_4_I.

From Figure 1b, a semi-crystalline feature of the CS polymer can be observed by two broad amorphous peaks centered at 2θ ranges from 33° to 45° and the pure CS exhibits several crystalline peaks at lower 2θ values [29]. Figure 2 provides insight into the PVA:CS blending system, indicating that there is no observable peak at 2θ =18.6°, which is the characteristic peak that appears for the pure PVA polymer. It is also observed that the hollow intensity decreases, and the broadening of line-width upon blending can be attributed to amorphous development [30].The XRD pattern for the PVA:CS blend electrolytes doped with various quantities of NH_4_I is exhibited in Figure 3. Obviously, it can be seen that as NH_4_I salt is increased up to 40 wt.% in the PVA:CS system, the hollow intensity substantially reduces and is accompanied by peak broadening. This indicates that the crystallinity of the blend polymer decreases at the expense of the amorphous region. At 50 wt.% of NH_4_I addition, several crystalline peaks appear as a result of recrystallization of the NH_4_I salt, and ion recombination could occur at high salt concentrations. It is worth mentioning that the crystalline regions in the polymer electrolytes decrease as a consequence of the complex formation. The complex formation results from the interaction between cations from the salt and the functional groups within the polar polymer backbone [31,32]. On the one hand, the periodic ordering of atom arrangements produces the lattice phases that are evidenced by scattering X-rays at certain directions [31]. On the other hand, the non-crystalline materials, such as the PVA:CS:NH_4_I system, are characterized by a distorted structure within the atomic arrangement in the unit cells, as presented in Figure 3a. This distortion causes various X-ray scattering in all directions, resulting in the appearance of broadened peaks as well as relatively low peak intensity [31,33]. It is of great importance that broad peaks appear in polymer electrolytes, which indicates an improvement in the ion conductivity. In addition, the dominance of the amorphous region accelerates segmental motion within the polymer backbone that, in turn, increases the ion migration and ion conductivity as investigated by Fan et al. [13]. Similarly, Rangasamy et al. [8] believed that an increase in the amorphous content of the polymer film results in enhancing ion mobility as a consequence of providing free volume in the polymer network. It also causes an increase in the segmental motion of the polymer chains, leading to an increase in the flexibility of the polymer matrix. Consequently, the overall ionic conductivity of the polymer electrolyte can be improved.

### 3.2. Morphological Study

**Figure 4:**
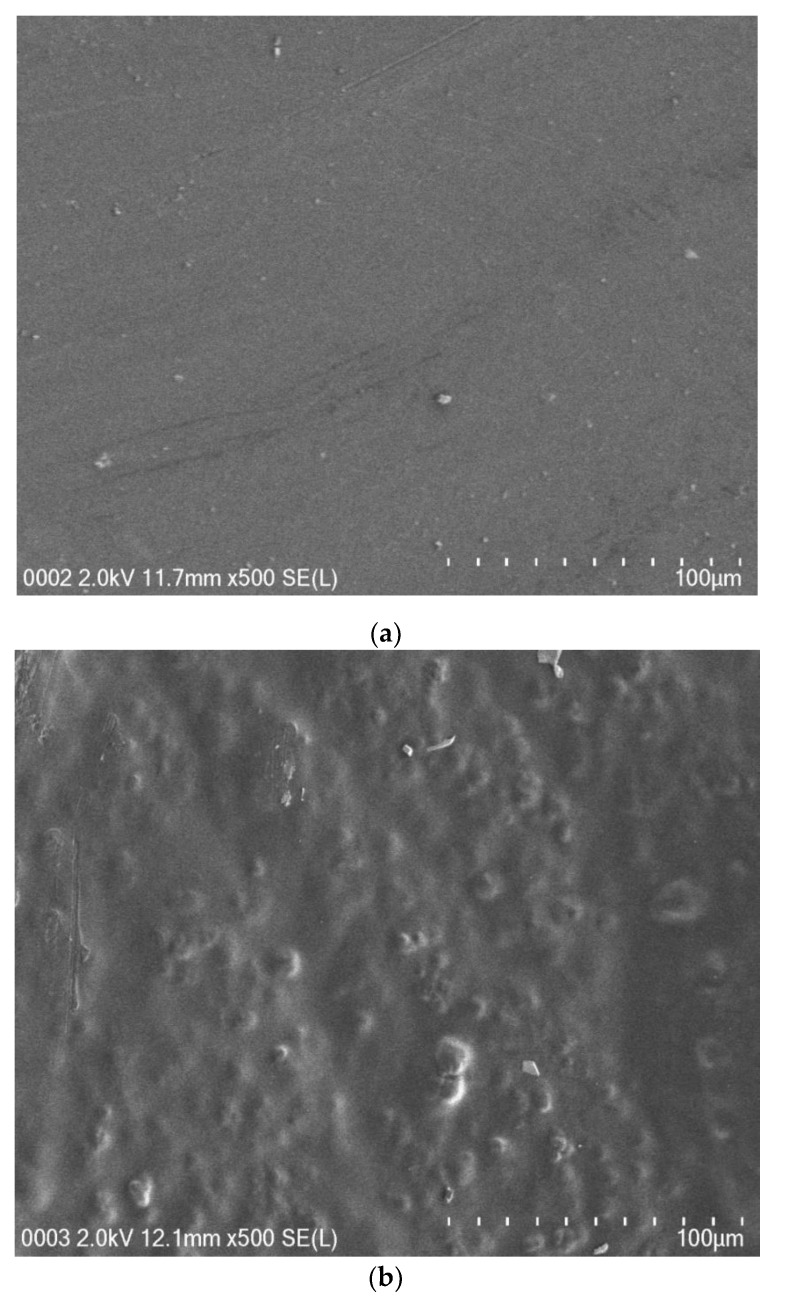

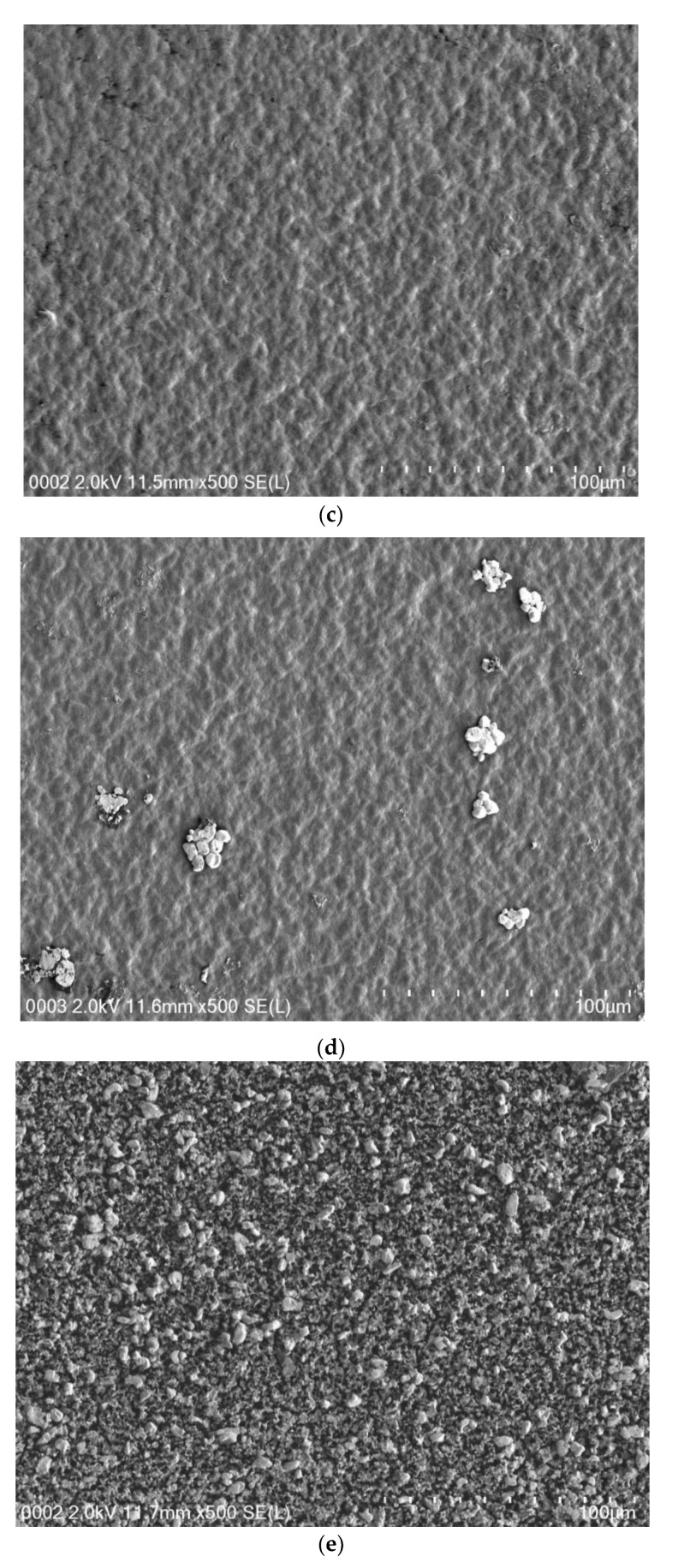
FESEM images for (**a**) PVCS 1, (**b**) PVCS 2, (**c**) PVCS 3, (**d**) PVCS 4 and (**e**) PVCS 5.

Figure 4a–e shows the SEM images of all SPE samples. It is clearly seen that the surfaces are relatively smooth to a large extent, in particular when the amount of NH_4_I salt up to 40 wt.% was incorporated. Earlier studies have shown that, from the surface morphology, one can obtain information about polymer/salt complexation and reduction of transition metal salts, such as silver salts or copper salts in polymer electrolytes [34,35,36,37]. It is also well documented that uniform surface morphology could be an indication of the forming of a membrane without porosity [38]. Mobarak et al. [39] showed that smooth surface electrolytes indicate moving ions more freely and thus increasing DC conductivity. Thus, from surface analysis, it is possible to gain insight into the changes in structural and electrical properties of polymer composite systems [34]. A. K. Arof and coworkers [40] successfully established a correlation between the surface morphology of SPEs and DC conductivity. The study showed that a drop in DC conductivity at relatively high salt concentration was recorded for CS:PVA:xNH_4_NO_3_ electrolyte systems. This can be explained by the fact that ion aggregations lead to the formation of protrusions on the surface. In our earlier study, a large number of protrusions on the film surface of a CS:NaTf system were obviously seen at 50 wt.% NaTf and they even covered almost the whole electrolyte surface [41].

### 3.3. Impedance and AC Conductivity Study

**Figure 5:**
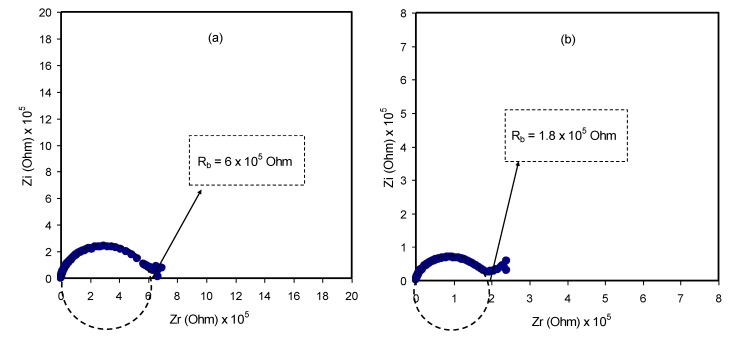

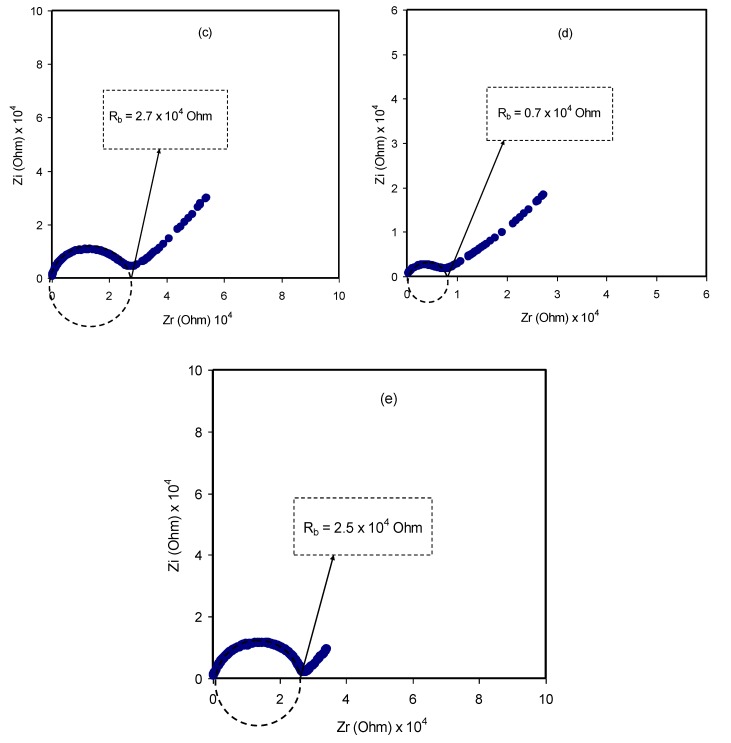
Complex impedance plots for (**a**) PVCS 1, (**b**) PVCS 2, (**c**) PVCS 3, (**d**) PVCS 4 and (**e**) PVCS 5.

The mechanism of ionic transport within polymer electrolyte systems is not fully understood, which becomes an obstacle to reaching the required ambient conductivity [25,42]. Nevertheless, impedance spectroscopy is a powerful technique to be used in the study of the ionic conductivity of polymer materials. Over the last decades, a new class of ion-conducting membrane materials has been the focus of researchers. Interest in these materials is returning to their wide use in solid-state electrochemical devices [43]. For this purpose, impedance spectroscopy was chosen to tackle the electrochemical properties of these materials, for instance, diffusion layer thickness, double-layer capacitance and charge transfer resistance [25]. From the impedance analysis, a plot for an ion-conducting polymer electrolyte is obtainable that consists of a small semicircle and a tail corresponding to high- and low-frequency regions, respectively. These responses provide insight into the bulk properties of the sample under study. It is observed that the depression of the semicircle size can be ascribed to the charge transfer at the interfacial region, as presented in Figure 5a–e. It is interesting to note that a capacitor-like material, a so-called pseudocapacitor, is formed at the sample/electrode interface resulting from double-layer growing [44]. A more interesting observation is the inclination caused by the blocking double-layer capacitance (i.e., electrode polarization) at the electrodes. The unparallel inclination of the straight line is other than the supposed value of 90° [45].

A direct relationship between the relatively high salt concentration and the bulk resistance can be established, as shown in Figure 5e. The impedance results are in strong agreement with the XRD results. The sharp peaks that correspond to the pure NH_4_I salt obviously appear to be shifted as a consequence of the ion association that lowers conductivity [30]. All these suggest that it is straightforward to detect ion association within electrolyte polymers using EIS. From the data analysis, the bulk resistance (*R_b_*) can be determined from the point where the real axis (*Z_r_*) and the semicircle intersect. To calculate the DC conductivity of the samples, the following relationship can be used using sample dimensions and the *R_b_* value [46]:

(6) $$\sigma_{dc}=\left(\frac{1}{R_{b}}\right)\times \left(\frac{t}{A}\right)$$

where *A* and *t* are the surface area and thickness of the films, respectively. The tabulated results of the computed DC conductivities are presented in Table 2. It is clear that the highest DC conductivity is obtained at 40 wt.% of NH_4_I and that the DC conductivity dropped as salt incorporation was introduced. The DC conductivity results are evidently in good agreement with the XRD results and surface morphology.

**Figure 6:**
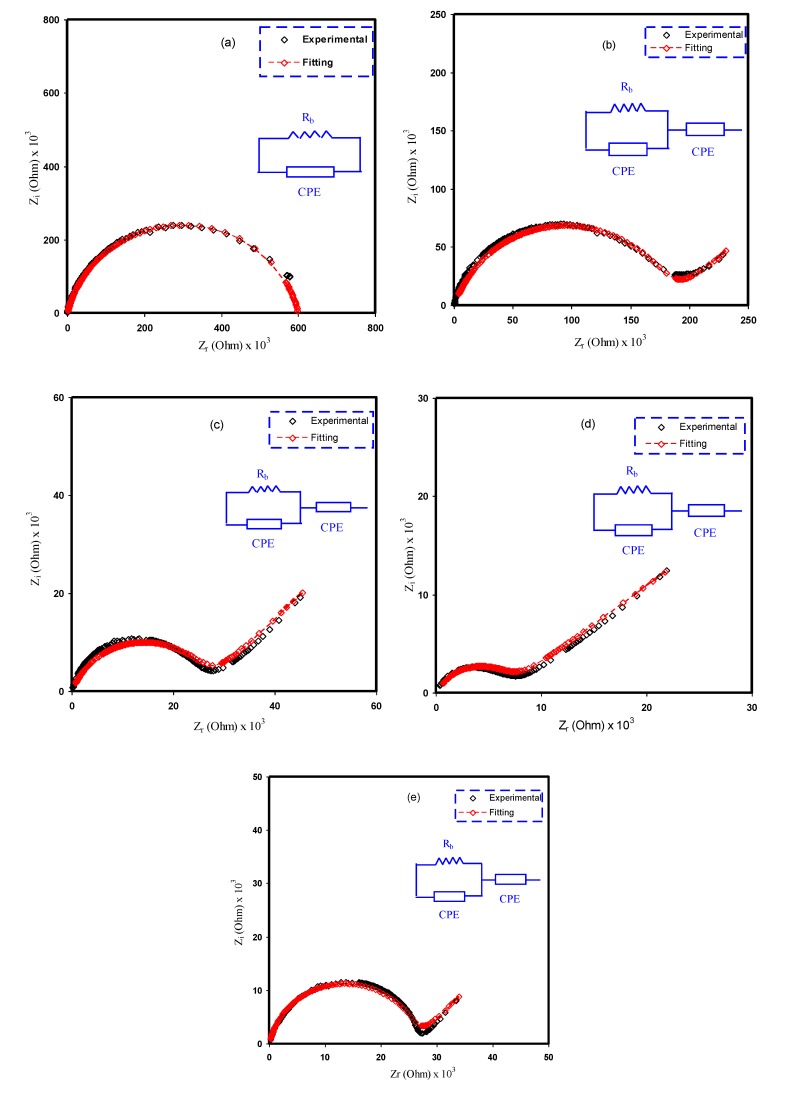
Experimental and EEC fitting plots for (**a**) PVCS 1, (**b**) PVCS 2, (**c**) PVCS 3, (**d**) PVCS 4 and (**e**) PVCS 5.

The electrical equivalent circuit (EEC) method is typically employed for examining EIS because the method is straightforward and rapid, as well as conveying a whole image of the PE system [3]. The Nyquist plot for the chosen PEs can be deduced with regard to the electrical equivalent circuit (EEC), comprising bulk resistance (*R_b_*) for the species charge carriers in the PE films as well as two constant phase elements (CPEs) as revealed in the inserts in Figure 6. The high-frequency area reveals the parallel connection of *R_b_* and CPE, while the low-frequency area displays CPE, specifically, the established EDLC between electrodes and electrolytes. The term CPE is more commonly used in EEC instead of ideal capacitor in the real system. The impedance of *Z_CPE_* can be written as follows [3]:

(7) $$Z_{CPE}=\frac{1}{C\omega^{p}}\left[\cos\left(\frac{\pi p}{2}\right)-i\sin\left(\frac{\pi p}{2}\right)\right]$$

where *C* denotes the *CPE* capacitance, *ω* stands for the angular frequency and *P* is linked to the divergence from the axis of the vertical EIS figures. Here, the real axis (*Z_r_*) and the imaginary axis (*Z_i_*) of the complex impedance (Z*) correlated with the EEC (insert in Figure 6a) can be demonstrated as follows:

(8) $$Z_{r}=\frac{R_{b}C_{1}\omega^{p1}\cos\left(\frac{\pi p_{1}}{2}\right)+R_{b}}{2R_{b}C_{1}\omega^{p1}\cos\left(\frac{\pi p_{1}}{2}\right)+R_{b}{}^{2}C_{1}{}^{2}\omega^{2p1}+1}$$

(9) $$Z_{i}=\frac{R_{b}C_{1}\omega^{p1}\sin\left(\frac{\pi p_{1}}{2}\right)}{2R_{b}C_{1}\omega^{{}^{p1}}\cos\left(\frac{\pi p_{1}}{2}\right)+R_{b}{}^{2}C_{1}{}^{2}\omega^{2P1}+1}$$

where *C*_1_ stands for the CPE capacitance at the bulk. Furthermore, the real axis (*Z_r_*) and the imaginary axis (*Z_i_*) of the complex impedance (Z*) correlated with the EEC (insert of Figure 6b–e) can be demonstrated as follows:

(10) $$Z_{r}=\frac{R_{b}C_{1}\omega^{p1}\cos\left(\frac{\pi p_{1}}{2}\right)+R_{b}}{2R_{b}C_{1}\omega^{p1}\cos\left(\frac{\pi p_{1}}{2}\right)+R_{b}{}^{2}C_{1}{}^{2}\omega^{2p1}+1}+\frac{\cos\left(\frac{\pi p_{2}}{2}\right)}{C_{2}\omega^{p2}}$$

(11) $$Z_{i}=\frac{R_{b}C_{1}\omega^{p1}\sin\left(\frac{\pi p_{1}}{2}\right)}{2R_{b}C_{1}\omega^{{}^{p1}}\cos\left(\frac{\pi p_{1}}{2}\right)+R_{b}{}^{2}C_{1}{}^{2}\omega^{2P1}+1}+\frac{\sin\left(\frac{\pi p_{2}}{2}\right)}{C_{2}\omega^{p2}}$$

where *C*_1_ stands for the CPE capacitance at the bulk and *C*_2_ stands for the CPE capacitance at the electrode/electrolyte interface.

Table 3 presents the fitting parameters in the EEC. The *R_b_* is moving away from the intersection of the semicircle or the line of the spike with the real part (see Figure 5). It is obvious from Figure 6 that, upon adding the salt, the semicircle at the high-frequency region becomes smaller up to 40 wt.% of the added salt.

**Figure 7:**
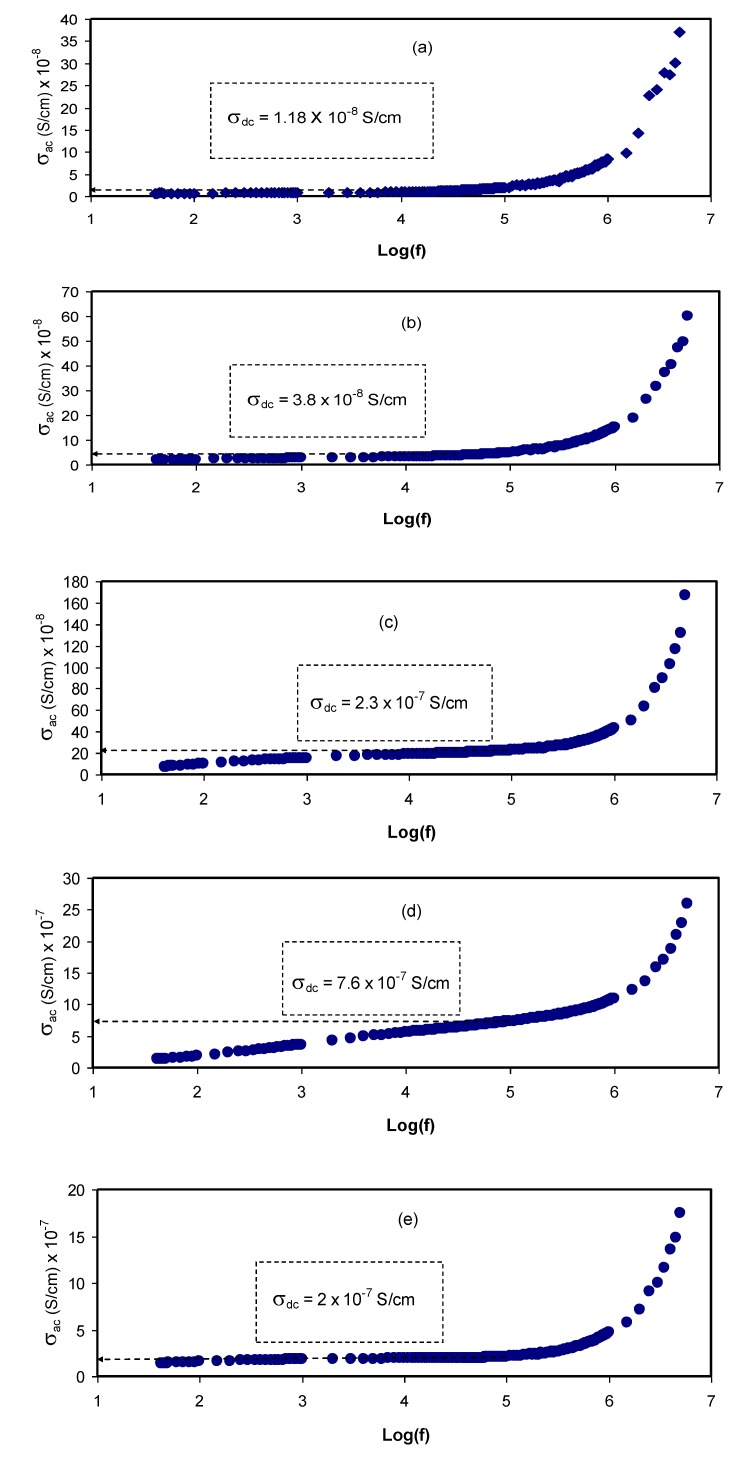
AC conductivity spectra for (**a**) PVCS 1, (**b**) PVCS 2, (**c**) PVCS 3, (**d**) PVCS 4 and (**e**) PVCS 5.

Figure 7 shows the AC conductivity spectra at room temperature for all the samples. The AC conductivities are calculated using the following relation [27]:

(12) $$\sigma {}^{\prime }{}_{ac}=\left[\frac{Z{}^{\prime }}{Z{}^{\prime }{}^{2}+Z^{{}^{\prime \prime }2}}\right]\times \left(\frac{t}{A}\right)$$

At a moderate salt concentration, three distinguished regions are seen, namely the low-frequency spike and the middle-frequency and high-frequency regions. At the low-frequency region, there is an electrode polarization that is directly proportional to the salt concentration. The middle-frequency region is seen almost in the form of a plateau that rises from the bulk DC conductivity. Interestingly, at the high-frequency region, the EIS response comes from conductivity relaxation that undergoes shifting as the salt concentration is increased [28]. An earlier study reported AC conductivity to the frequency of the electric signal correlation, which was then used in the analysis of electrical DC conductivity [35].

From this finding, it is possible to determine the DC conductivity by extrapolating the plateau region on the *y*-axis of the AC conductivity spectra. It is worth noting that the DC conductivity values (see the insets in Figure 7) are quite close to those obtained from the bulk resistance (*R_b_*), as presented in Table 2. It is interesting to note that as the salt concentration is increased, the dispersion region becomes narrow. This is directly governed by electrode polarization (in the form of spikes). It is also seen that the AC conductivity increases as it moves toward the higher frequency region. From this increase in AC conductivity with frequency, one can suggest that the hopping conduction mechanism occurs where charge carrier hopping is enhanced between the localized states [47]. These observations in the study of AC conductivity have been confirmed in earlier work [27]. Analyzing AC conductivity spectra, it is easy to determine DC conductivity. It is also possible to compare the impedance spectra to show a strong relationship between the dispersion region of AC conductivity and the high-frequency semicircle (see the Nyquist plot in Figure 5).

The presence of a spike region is the response of adding a relatively high amount of salt, as shown in Figure 7. The phenomenon of AC conductivity results from charge carrier confinement throughout the whole body of the sample [27,47]. Based on the well-known Jonscher universal power low, specifying the nature of ion dynamics by calculating the frequency exponent (s) can be accurately obtained as follows [26,27,28]:

*σ^′^_ac_(ω) = σ_dc_ + A ω^s^*    (0 < s <1), (13)

For most ion conductors, the second term of Equation (8) (i.e., σ′_ac_(ω) = A ω*^s^*) is followed by the dispersion region of AC conductivity where the frequency exponent (*s*) is less than unity [16]. It is also important to examine the first term, which is related to the plateau response of the AC spectra, and its extension to the *y*-axis can be used to estimate DC conductivity [26,28]. The DC conductivity values obtained from the AC spectra are presented in Table 2. It is noteworthy that it is of vital importance to show a good agreement between DC conductivity obtained from the AC spectra and the impedance results.

### 3.4. Study of Dielectric Properties

**Figure 8:**
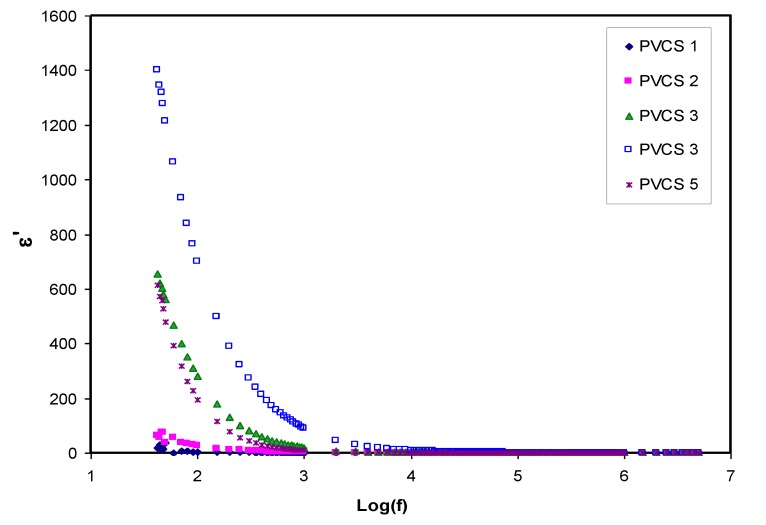
Dielectric constant versus log (f) for all polymer blend electrolytes.

**Figure 9:**
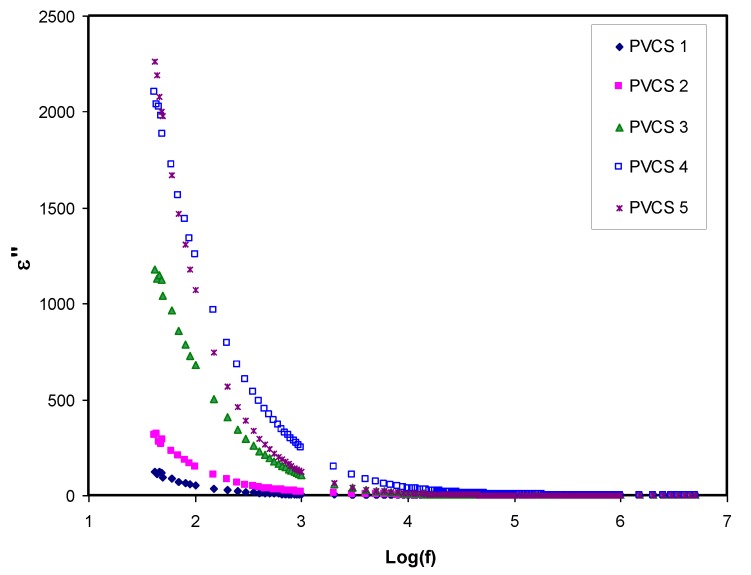
Dielectric loss versus log (f) for all polymer blend electrolytes.

Dielectric constant analysis is an informative way to deal with the mechanism of ion transportation and the phase transitions within polymer electrolytes. It is well known that ion pairs, triplets and clusters cause a lowering of electrical conductivity. It is important to mention that ion pairs possess a higher permanent dipole moment compared to others. Importantly, the host polymer shows a relatively low dielectric constant despite the existence of ion pairs when the dielectric measurement is performed [25,48,49,50,51,52].Figure 8 and Figure 9 explain the variation of dielectric constant and dielectric loss for all blended electrolyte samples at ambient temperature. It can also be seen that both dielectric parameter (ε′, ε″) values are relatively high in the low-frequency region, indicating the electrode polarization phenomenon. This phenomenon results from local charge accumulation at the electrode/electrolyte interface region. More significantly, this phenomenon is the result of the difference in conductivity between two materials in contact with each other [46]. An increase in salt concentration results in an increase in dielectric constant and dielectric loss of the PVA:CS solid polymer electrolyte. This can be correlated to both the bond energy of the salt and the polymer and an increase in polarization [28,49]. In general, the stable structure of the polymer comes from two main forces, namely primary intra- and secondary intermolecular forces [50]. The primary forces comprise covalent bonding (2.2–8.6 eV) and ionic bonding (0.43–0.87 eV) that connect atoms in the polymer backbone chain, whereas the secondary forces are hydrogen bonding (0.13–0.30 eV), dipolar interactions (0.07–0.13 eV) and dispersion interactions (0.002–0.09 eV). In terms of dissociation energy, it is easy to break down the secondary forces compared to the primary ones. Based on this explanation, the secondary forces can easily be disrupted with salt addition that, in turn, impacts considerably on the polymer segmental motions within the polymer body. In other words, salt addition affects the dielectric behavior, charge transport and charge storage. As a consequent, a double-layer capacitance can develop from ion transport that accumulates between the sample and the electrodes. In addition, as the applied field frequency increases, the available drift time reduces. Accordingly, the dielectric constant decreases, and the required time for charge carrier drifting reduces; therefore, both real and imaginary parts of the dielectric properties decrease [51,52,53]. Principally, polarization results from charge orientation and ultimately disappears due to the inertia of the ions [49]. It is clear that a high dielectric constant is obtained for 40 wt.% of NH_4_I addition and then it drops in the case of 50 wt.% because of electrode/electrolyte interface blocking. This is further confirmed by the DC conductivity values presented in Table 2. The physics of this DC conductivity and dielectric constant relationship can be qualitatively explained. In general, the famous expression for conductivity is formulated by the following:

*σ* = ∑*nqμ* (14)

where *n* is the charge carrier density, *q* is 1.6 × 10^−19^ C and *μ* is the mobility of the ions. Based on this formulation, it can be clearly seen that both ionic mobility and the number of charge carriers increase as the salt concentration is increased in the range 10 to 40 wt.%. However, further salt addition leads to a decrease in dielectric parameters, for example, at 50 wt.%. Moreover, the carrier density (i.e., number of the charge) is directly related to the bond dissociation energy *U* and dielectric constant ε′, which can be understood via this relationship (*n* = *n*_o_ exp(*−U/*ε′*KT*)). For example, the addition of salts into polymer matrices causes an increasing dielectric constant, as a consequence of increasing the charge carrier density [54]. Therefore, to determine the conductivity behavior of the solid polymer electrolyte, relationship between DC conductivity and dielectric constant must be established as well as to salt concentration at room temperature.

**Figure 10:**
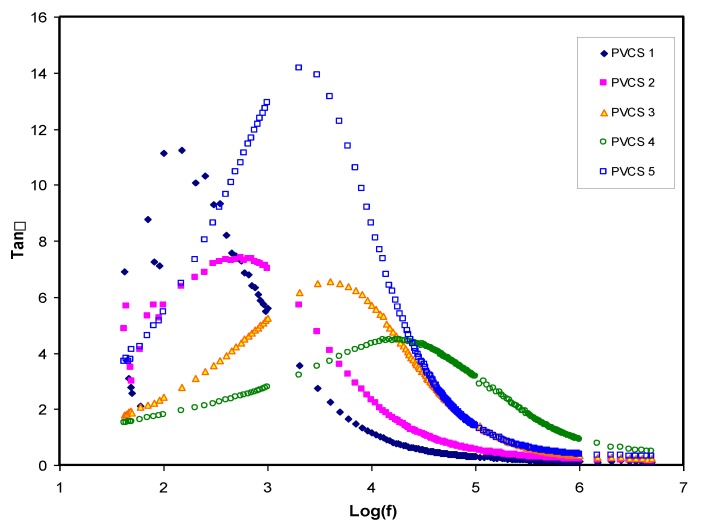
Tanδ versus log (f) for all polymer blend electrolytes.

The relaxation processes of polymer materials can be accurately examined via loss tangent peaks. The dipoles in the polymer electrolytes can certainly be interpreted on the basis of the dielectric relaxation. Figure 10 shows dielectric relaxation peaks of the loss tanδ versus frequency plot for all PVCS electrolyte samples at room temperature [52]. In the figure, there is a shift to a higher frequency region of the loss tangent peak, indicating the occurrence of dielectric relaxation. Furthermore, the shortest relaxation time of the PVCS4 electrolyte sample can be observed via the loss tangent plot. One finding of this study is that permanent or induced dipoles cause the electric conductivity and dielectric relaxation peaks. It has also been observed that induced diploes hidden the polarization relaxation of mobile charged species in the materials [55]. Based on Koops phenomenological model, the loss tangent shape can be interpreted [56]. Accordingly, the low-frequency dispersion curve possesses a negative slope, suggesting the loss of dominancy of conduction and that it is modeled via a parallel RC circuit in the adopting homogeneous system. The EECs presented in Figure 7 support precisely the Koops model. The loss tangent intensity increases to a maximum at a certain frequency value and then decreases as the frequency increases. This is related to the fact that active component (ohmic) of the current increases more rapidly than the reactive component (capacitive). More than one relaxation process causes the loss tangent peaks to be broad and to obey non-Debye type relaxation [57]. As previously shown, the tanδ plot is helpful in calculating transport parameters, namely the diffusion coefficient, carrier density and mobility [58].

**Figure 11:**
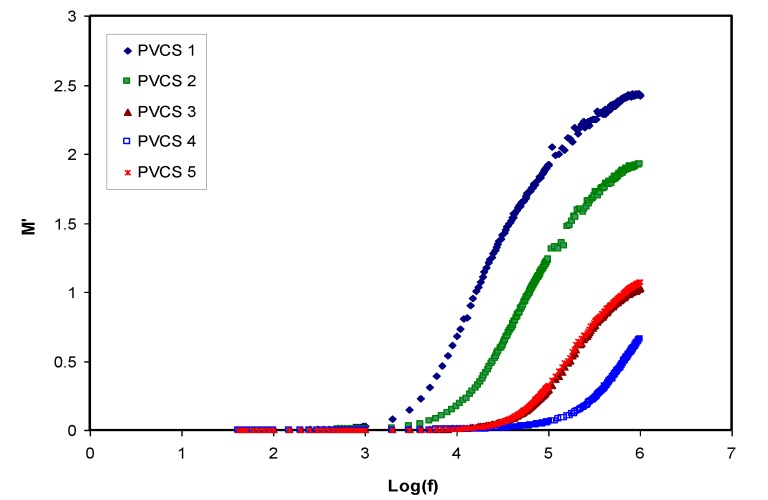
Real part of electric modulus versus log (f) for all polymer blend electrolytes.

**Figure 12:**
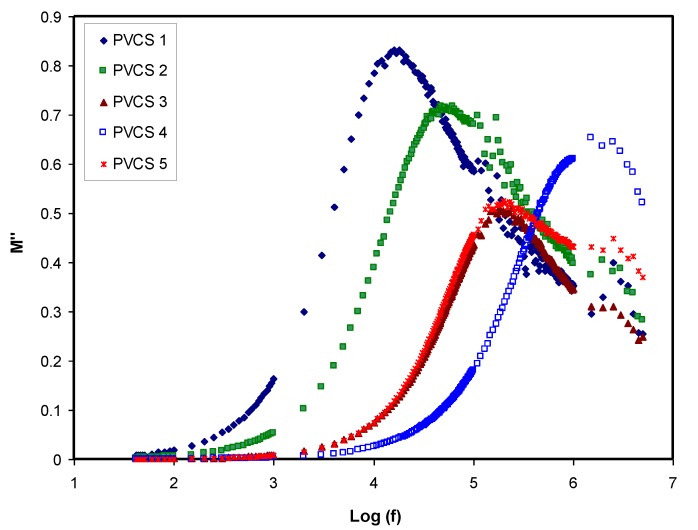
Imaginary part of electric modulus versus log (f) for all polymer blend electrolytes.

Conductivity and relaxation dynamics are frequency dependent and are responsible for charged species motion and the induction of dipoles in the polymer electrolyte. The relaxation dynamics from dielectric relaxation can be investigated using electric modulus formalism [52,59]. To obtain bulk relaxation properties, the electric modulus is helpful. The electric polarization (EP), space charge injection phenomena and conduction effects can be understood via electric modulus formalism [27,60]. The evaluations of the real and imaginary parts of the electric modulus were carried out using Equations (4) and (5). Figure 11 and Figure 12 exhibitthe real and imaginary parts of the electric modulus versus frequency for all PVCS electrolyte samples at room temperature. At low frequency, a long tail for either *M′* and *M″* is seen and is related to the capacitance obtained from the double-layer charge building up at the interfacial region [46,59]. In comparison, the ε′ and ε″ spectra and the *M′* and *M″* behave in exactly the opposite manner. It is interesting that the dielectric constant’s high value (see Figure 8) is seen at the low-frequency region. Based on the principles, the electric moduli (*M′* and *M″*) are made from the reciprocal of the complex dielectric constant, recording a minimum value at high frequency [61]. From the unusual response of *M″*, it is difficult to apply simple exponential Debye to interpret the relaxation process. It is extremely interesting to note the relaxation peak appearance in the *M″* spectra (see Figure 12) and the disappearance in the dielectric loss spectra, as exhibited in Figure 9. The appearance of peaks in the imaginary part of the modulus spectra indicates the existence and contribution of huge amounts of electrode polarization in the dielectric loss parameter. This can be used in tackling the conduction process mechanism in polymer electrolytes, where it progresses via ion migration between coordinated sites within the polymer body and segmental relaxation. In other words, the appearance of peaks in the *M″* spectra can be interpreted as a result of a couple of species motions, namely ionic and polymer segmental motions [62,63]. An interesting observation can be seen from the peak shifting of relaxation to the lower frequency side with an increase to 50 wt.% of NH_4_I salt. This reveals a strong relationship between relaxation time and salt concentration, which is directly proportional. Basically, it has been documented that the relaxation time increases as the ionic mobility decreases [57]. Specifically, the relaxation time decreases as conductivity increases, as reported in previous work [52,58,59,64]. In the current study, it was confirmed that the relaxation time increases when conductivity decreases.

### 3.5. Electrochemical Characteristics

#### 3.5.1. TNM Measurement 

**Figure 13:**
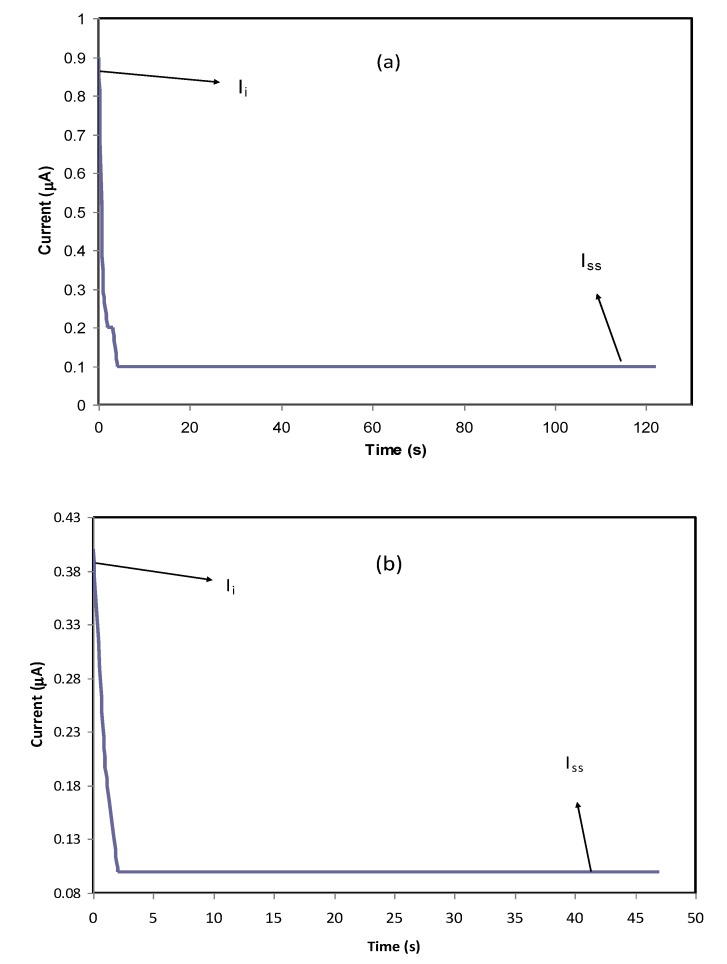
Polarization current versus time for (**a**) PVCS4 and (**b**) PVCS5 electrolyte films.

**Figure 14:**
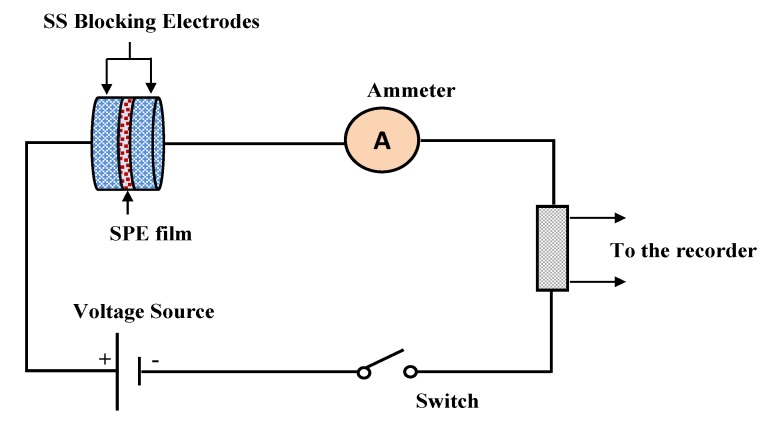
TNM measurement setup.

The heart of electrochemical devices is considered to be the ion-conducting electrolyte, for instance, in batteries and supercapacitors; thus, the study of electrolytes has priority [65]. The focus has been devoted to evaluating the contribution of both ions and electrons in the polymer electrolyte and metallic solid materials, respectively. It is clearly known that, at the interfacial region, charge transfer occurs between two different phases, i.e., the solid metallic electrode and the liquid (electrolyte) phases. It is of significant importance to determine the identity of species that are responsible for carrying a charge in the system under study. Herein, a 0.8 V of the DC polarization method was applied to the working electrode, and transfer number measurement (TNM) was performed. The information obtained about the TNM plots for PVCS4 and PVCS5 electrolyte samples from the polarization responses at room temperature versus time can be seen in Figure 13a,b. The nature of ion transport was evaluated via Wagner’s polarization method in an attempt to calculate the total ionic transference number (*t_ion_*) from the current–time plot. From the plot, there is a sudden drop in current within the system at the beginning, indicating the extent of contribution to conductivity by ions versus electrons [12]. The values of *t_ion_* and *t_el_* were estimated using the following equations [66]:

(15) $$t_{ion}=\frac{I_{i}-I_{ss}}{I_{i}}$$

(16) $$t_{el}=1-t_{ion}$$

where *I_i_* and *I_ss_* are the initial current and the steady-state current, respectively. From the TNM plots, as the potential is swept, at the beginning stage, there is a huge current rise of 0.89 µA and 0.41 µA for PVCS4 and PVCS5, respectively. This results from the contribution of both electrons and ions in the conduction. The value of ion transport *t_ion_* was extracted from the initial and steady-state currents of the PVCS4 and PVCS5 samples, and found to be 0.88 and 0.75, respectively. It can be seen that the value of *t_ion_* of PVCS4 is higher than PVCS5. The general response profile consists of three distinct regions, namely the initial current rise, current decay and steady-state current. At the initial state, the current flows across the cell at the blocking electrode under the impact of an applied voltage are due to electrode charging. In other words, at the initial stage of the electrochemical course, the DC potential results in the current creation that is proportional to both the ion migration and electron moving. The electric field encourages mobile species to be pushed to move in the electrolyte as a result of the migration of ions. Subsequently, the current drops within a short time period, where ion drifting is equivalent to ion diffusion. At steady state, the diffusion layer develops from a concentration gradient induced by electrode polarization at the interface region where polarization occurs for a long period of time. Additionally, the DC polarization at the interfacial region faces a substantial resistance from the passive layer formation by ions. This means the current comes solely from electrons, i.e., the entire current is from electrons with no contribution by ions. Based on the ion transfer number values of 0.88 and 0.75 of the two samples, the main contributor of the carrying charge are ions rather than electrons [67]. Therefore, from the TNM measurement it can be concluded that PVCS4 is a preferable system for electrochemical device applications, in particular if the conductivity of the system is maximized to 10^−3^–10^−4^ S/cm. The TNM measurement setup is shown in Figure 14. The initial current is very important, and as the cell is subjected to 0.2 V, the first current response in the multimeter during the switch-on was taken as initial current. The initial current is high due to the contribution of both ions and electrons.

#### 3.5.2. Linear Sweep Voltammetry (LSV) Studies

**Figure 15:**
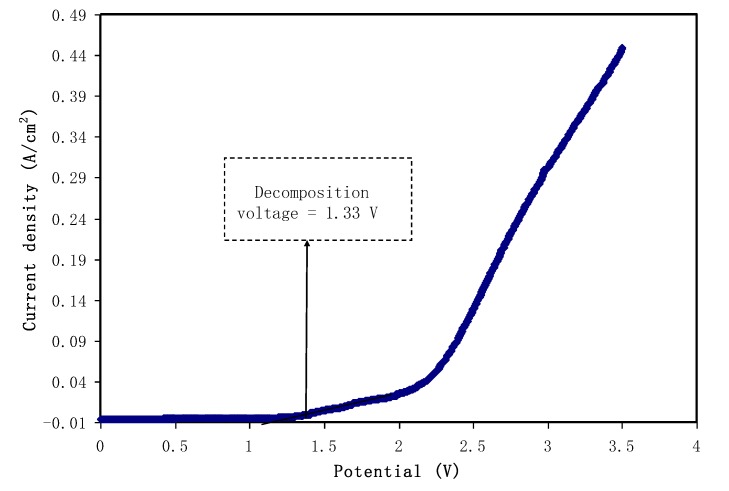
Linear sweep voltammetry (LSV) plot for the highest conducting (PVCS4) blend electrolyte film.

In order to determine the electrochemical stability of the polymer electrolyte under study, linear sweep voltammetry (LSV) is a straightforward and powerful technique to use. LSV was performed for the highest DC conductivity and TNM value sample.The LSV response of the polymer electrolyte PVA:CS:NH_4_I (PVCS4) at 10 mV/s sweep rate with room temperature is shown in Figure 15. It can be seen that the current rose considerably and sharply at 1.33 V, which indicates the electrolyte decomposition. This potential cut-off from the LSV response suggests the eligibility of the electrolyte for use in proton-based energy devices. For comparison, this result is quite close to that documented for a polymer electrolyte based on ammonium salt found in the literature. Ng and Mohamad reported the cut-off potential of 1.8 V for a chitosan-based membrane made of ammonium nitrate salt and ethylene carbonate plasticizer at room temperature [68]. In another study conducted by Kadir et al., the potential window of 1.7 V was reported for CS:poly(vinyl alcohol) (PVA) [69]. Comparably, in our earlier report on PVA:dextran:NH_4_I system, 1.3 V was obtained [70]. There is harmony between the potential window that is lower than 1 V and undesired consequences, such as solvent evaporation and leakage in supercapacitor systems [10]. Consequently, these results help to decide on the eligibility of PVA:CS:NH_4_I as the electrolyte of choice in EDLC applications.

## 4. Conclusions

In conclusion, the preparation and characterization of polymer blend electrolytes based on poly(vinyl alcohol):chitosan (PVA:CS) polymers were studied. The electrical characterizations (ε′, ε″, tangδ, M′ and M″) were carried out in order to understand that ion transport occurs through the coupling between ion and polymer segmental motion. The electrochemical investigations (TNM and LS) were performed in order to examine the suitability of the samples for energy storage device applications. The structural analysis from X-ray diffraction (XRD) revealed the structural change upon the addition of NH_4_I salt. As the salt concentration increased to 50 wt.% NH_4_I, several crystalline sharp peaks were observed in the XRD spectra due to the salt’s protruded appearance. The protruded appearance on the samples’ surface was evidently shown at high salt concentrations in the field-emission scanning electron microscope (FESEM) images. The XRD and FESEM results support each other. The optimum salt content is 40 wt.% of NH_4_I to reach the maximum DC conductivity (9.1 × 10^−7^ S/cm). The most amorphous system exhibits the highest DC conductivity. To obtain further insight into the electrical characteristics of the ion-conducting films, the EIS spectra were fitted with electrical equivalent circuits (EECs). The conductivity measurements of the samples were correlated with their dielectric properties. At the low-frequency region, high values of dielectric constant and dielectric loss were observed due to electrode polarization. From loss tangent and electric modulus plots, the broad nature of the peaks appeared in the tanδ and imaginary parts of the electric modulus, indicating the distribution of relaxation times. From the TNM measurements, both ionic (*t_ion_*) and electronic (*t_ele_*) transference numbers were evaluated. It was found that the *t_ion_* values for PVCS4 and PVCS5 samples were 0.88 and 0.75, respectively. This indicates that the system incorporated with 40 wt.% of NH_4_I salt exhibits a high ion transference number. A potential cut-off of 1.33 V was recorded for the electrolyte system as decomposition voltage. This allowed us to conclude that the PVA:CS biopolymer electrolyte in this work is electrochemically stable up to above 1 V, which is important for use in electrical double-layer capacitor (EDLC) applications.

## Figures and Tables

**Table 1:** Designation and composition of the various PVA:CS:NH_4_I solid polymer electrolytes (SPEs).

Sample Designation	(PVA:CS)(0.5:0.5) (g)	NH_4_Iwt.%
PVCS1	1	10
PVCS2	1	20
PVCS3	1	30
PVCS4	1	40
PVCS5	1	50

**Table 2:** DC conductivity for PVA:CS:NH_4_I blend electrolyte films from Equation (6) and AC conductivity spectra at room temperature.

Sample Designation	DC Conductivity (S/cm) (Using Equation (6))	DC Conductivity (S/cm) (Using AC Plot)
PVCS1	1.13 × 10^−8^	1.18 × 10^−8^
PVCS2	3.77 × 10^−7^	3.8 × 10^−7^
PVCS3	2.51 × 10^−7^	2.3 × 10^−7^
PVCS4	9.71 × 10^−7^	7.6 × 10^−7^
PVCS5	2.72 × 10^−7^	2.0 × 10^−7^

**Table 3:** Fitting parameters of the EEC for electrolyte films at room temperature.

Sample	p1(rad)	p2(rad)	C_1_(F)	C_2_(F)
PVCS1	0.859	-	1.43 × 10^−10^	-
PVCS2	0.71	0.8	3.33 × 10^−10^	1.05 × 10^−6^
PVCS3	0.8	0.5	2.00 × 10^−9^	2.00 × 10^−6^
PVCS4	0.78	0.42	5.00 × 10^−9^	4.35 × 10^−6^
PVCS5	0.89	0.52	1.00 × 10^−9^	4.17 × 10^−6^
